# Chemical genetics to examine cellulose biosynthesis

**DOI:** 10.3389/fpls.2012.00309

**Published:** 2013-01-29

**Authors:** Chad Brabham, Seth DeBolt

**Affiliations:** Plant Physiology, Department of Horticulture, University of KentuckyLexington, KY, USA

**Keywords:** chemical genetics, small molecules, cell wall, cellulose, microtubules, isoxaben, DCB, CESA

## Abstract

Long-term efforts to decode plant cellulose biosynthesis via molecular genetics and biochemical strategies are being enhanced by the ever-expanding scale of omics technologies. An alternative approach to consider are the prospects for inducing change in plant metabolism using exogenously supplied chemical ligands. Cellulose biosynthesis inhibitors (CBIs) have been identified among known herbicides, during diverse combinatorial chemical libraries screens, and natural chemical screens from microbial agents. In this review, we summarize the current knowledge of the inhibitory effects of CBIs and further group them by how they influence fluorescently tagged cellulose synthase A proteins. Additional attention is paid to the continuing development of the CBI toolbox to explore the cell biology and genetic mechanisms underpinning effector molecule activity.

## INTRODUCTION

A chemical inhibitor approach utilizes bioactive small molecules instead of genetic lesion to disrupt protein function and have been applied to answer many fundamental questions in plant science (Zhao et al., 2003; Armstrong et al., 2004; Surpin et al., 2005; Rojas-Pierce et al., 2007; Bassel et al., 2008; De Rybel et al., 2009; Park et al., 2009; Santiago et al., 2009; Ovecka et al., 2010; Drakakaki et al., 2011). There are some exploitable differences between chemical and traditional genetics. Small molecules can be employed to help circumvent lethal loss-of-function mutations. Alternatively, an inhibitor can overcome genetic redundancy that results in masking of the mutant phenotype by targeting a clade of common gene products with a single mechanism of action (Robert et al., 2009; Toth and van der Hoorn, 2009). However, challenges can arise with compounds that display broad inhibitor activity on a large class of structurally similar proteins that function in subtly different ways or where the mechanism of action has not fully been elucidated making it difficult to appropriately interpret plant response. In an ideal setting a small molecule can provide experimental flexibility allowing for use at precise temporal points for rapid, yet reversible inhibition of a target pathway.

Drug dose rates are generally tuneable, which allows for a range of phenotypes to be observed over various concentrations. For example, a tuneable gradient could be used to generate a dose that barely compromises or completely inhibits growth. The mid range dose, named the lethal dose 50 (LD_50_). This tuneable nature of inhibitors can then be combined with mutagenesis studies in plants to isolate mutants that are resistance to the LD_50_ or hypersensitive to a dose that barely compromises plant growth. The hypothesis is that a resistant or hypersensitive mutant will provide new genetic elements involved in a target pathway. Examples of this type of experimental design will be referred to for cellulose biosynthesis inhibition. The overarching challenge has been to isolate a genetic mutation that confers resistance in an ethyl methane sulfonate treated population, which are often missense mutations. Map-based cloning is then needed, which traditionally required hundreds if not thousands of segregating individuals (Scheible et al., 2001). With the advent of next-generation sequencing it is now feasible to map single base pair mutations using a small number of homozygous individuals within a mapping population (around 20). This will reduce the raw material requirements of map-based cloning efforts to hours rather than months (see Vidaurre and Bonetta, 2012 for further information). Moving from a drug-induced phenotype to a genetic component required a substantial resource investment. As we review herein, the use of cell biology to examine cellulose biosynthesis inhibitors (CBIs) has been a valuable intermediary that allows the researcher to explore the mechanism by which cellulose synthase A (CESA) responds to the drug, and secondly learn more about CESA behavior in living cells. The current mini-review provides an overview of the developing toolbox of compounds that perturb cellulose biosynthesis.

## CHEMICAL GENETICS TO DISSECT CELLULOSE BIOSYNTHESIS

In plants, anisotropic cell growth is facilitated by a rigid, yet extensible cell wall, which acts to collectively constrain internal turgor pressure. Cellulose forms the central load-bearing component of cell walls and is necessary for plant cell expansion. Hence, inhibiting cellulose biosynthesis causes radially swollen tissues in seedlings providing a robust phenotype for genetic screens. In contrast to the Golgi-fabricated hemicellulose and pectin carbohydrate units in the cell wall matrix, plants synthesize cellulose at the plasma membrane by a globular, rosette-shaped, protein complex, collectively referred to as cellulose synthase complex (CSC; Mueller and Brown, 1982; Haigler and Brown, 1986; Brown, 1996). The CSC contains a number of structurally similar CESA catalytic subunits (Pear et al., 1996; Saxena and Brown, 2005) that extrude *para*-crystalline microfibrils. Microfibrils are made up of multiple, unbranched, parallel (1,4) linked β-D-glucosyl chains. The predicted membrane topology of a typical plant CESA has a cytoplasmic N-terminal region with a zinc-finger domain followed by two transmembrane domains (TMDs), a large cytoplasmic domain containing the catalytic motifs, and finally a cluster of six TMDs at the C-terminus. Hypothetical models based on this topology suggest that eight TMDs anchor the monomeric protein in the plasma membrane and create a pore through which a polymerizing glucan chain extrudes (Delmer, 1999).

Experimental evidence for the dynamic behavior of CESA in living plant tissue has arisen via the use of live-cell imaging (laser spinning disk confocal microscopy; Paredez et al., 2006). Transgenic *Arabidopsis* plants carrying a fluorescent protein reporter on the N-terminal of CESA6 or CESA3 have demonstrated quantifiable behaviors of the CSC at the plasma membrane such as relatively constant velocity of the CSC at the plasma membrane focal plane (∼250 nm.min^−1^). Furthermore, the presence of the CESA reporter has been aligned with a suite of intercellular compartments (Paredez et al., 2006; Crowell et al., 2009; Gutierrez et al., 2009). Examination of CESA behavior in combination with CBI treatments can provide a platform to ask questions of the cell biology and will be examined herein. Unfortunately, plant CESA proteins have not been crystallized, nor has a functional CSC been purified *in vitro*, therefore the precise associations between CBIs and CESA are correlative. Nevertheless, the use of these inhibitors, as detailed below, has been of use in obtaining rational theories regarding the mechanism of delivery, activation, movement, and array organization during cellulose biosynthesis.

## CLASSIFYING INHIBITOR PHENOTYPES ON CESA IN LIVING TISSUE

**FIGURE 1:**
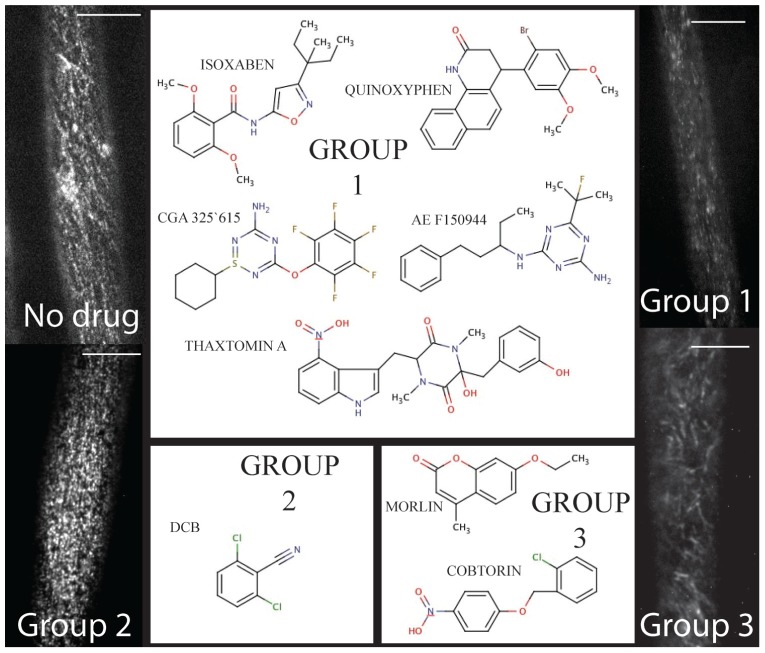
**The chemical toolbox for dissecting cellulose biosynthesis via live-cell imaging.** Group 1 includes compounds such as isoxaben and thaxtomin A that induce clearance of CESA from the plasma membrane. By contrast, Group 2 is comprised of DCB, which causes a syndrome of reduced CESA velocity and hyperaccumulation at the plasma membrane. Finally, Morlin and cobtorin (Group 3) induce the plasma membrane localized CESA to move with aberrant trajectory and cause reduced CESA movement. For each example, the scale bar = 10 μm (images courtesy of Seth DeBolt).

Three principle responses to chemical inhibition have been documented via live-cell imaging thus far, and inferences can be made beyond live-cell imaging to cluster compounds into similar response groups. Each of the response phenotypes will be discussed independently below and are broadly summarized as (1) clearing of CESA from the plasma membrane focal plane, (2) stopping the movement of CESA, and (3) modifying the trajectory of CESA to or in the plasma membrane (**Figure 1**). Other CBI compounds have been characterized, but experiments with real-time confocal imaging of fluorescently tagged CESA have not been performed and are not discussed, accordingly.

## CESA CLEARING FROM PLASMA MEMBRANE

The first group includes compounds that deplete the CSC from the plasma membrane (**Figure 1** – Group 1). CBIs in this group include isoxaben (N-[3-(1-Ethyl-1-methylpropyl)-5-isoxazolyl]-2,6-dimethyoxybenzamide), thaxtomin A ((4-nitroindol-3-yl-containing 2,5-dioxopiperazine), AE F150944 (N2-(1-ethyl-3-phenylpropyl)-6-(1-fluoro-1-methylethyl)-1,3,5-triazine-2,4-diamine), CGA 325’615 (1-cyclohexyl-5-(2,3,4,5,6-pentafluorophenoxyl)-1λ,2,4,6-thiatriazin-3-amine), and quinoxyphen (4-(2-bromo-4,5-dimethoxy-phenyl)-3,4-dihydro-1H-benzo-quinolin-2-one) (Paredez et al., 2006; Bischoff et al., 2009; Crowell et al., 2009; Gutierrez et al., 2009; Harris et al., 2012). All of the compounds are synthetically derived, except for thaxtomin A, which is a phytotoxin produced by *Streptomyces* species pathogenic to potato and other taproot crops (Scheible et al., 2003). Forward genetic screens have identified point mutations that confer resistance to isoxaben in CESA3 and CESA6 (Heim et al., 1989; Scheible et al., 2001; Desprez et al., 2002), and quinoxyphen-resistance in CESA1 (Harris et al., 2012). This data further supports the notion that CESA1, 3, and 6 interact to form a functional CSC required for primary cell wall biosynthesis, since both compounds affect YFP-CESA6 similarly in susceptible seedlings (Baskin et al., 1992; Persson et al., 2007; Gutierrez et al., 2009; Harris et al., 2012). Moreover, quinoxyphen-resistance mutation was mapped to Ala903Val in *A. thaliana* CESA1, which has recently been aligned with Tyr455 in TMD6 of BCSB (Morgan et al., 2012). These authors demonstrate that Tyr455 forms a hydrogen bond to the translocating glucan during cellulose synthesis. Thus, quinoxyphen-resistance mutations are consistent with quinoxyphen action being inhibition of translocation rather than catalysis during cellulose biosynthesis.

Subsequent live-cell imaging (>20 min) after aforementioned drug treatment reveals that the plasma membrane eventually is devoid of CESA and fluorescently labeled CESAs accumulate in static and/or erratically moving cytosolic CESA containing compartments (SmaCC/MASC; Crowell et al., 2009; Gutierrez et al., 2009). Several possible scenarios may result in the clearance phenotype. For instance, the activity of the CBI leading to CESA depletion from the plasma membrane might modify vesicular trafficking and stop CESA cargo from reaching the site of synthesis. Further, CBI activity could target many processes in the endomembrane system, changing the speed of cycling, or modify CESA localization. It is also not possible to rule out that depletion of CESA from the plasma membrane is the result of natural protein turnover (∼*Gh*CESA1 half life < 30 min; Jacob-Wilk et al., 2006). Alternatively, drug treatment could cause disassembly of CSCs and induce CESA endocytosis. For instance, freeze-fracture images of AE F150944 treated *Z. elegans* tracheary elements provide data showing that the few detectable plasma membrane rosettes are destabilized (control diameter 24 nm vs treated 30 nm; Kiedaisch et al., 2003). Decoding how and why CESA is cleared from the plasma membrane is a keenly awaited result.

Cellulose biosynthesis inhibitors that clear the plasma membrane of CESA may be used to monitor non-CESA proteins associated with cellulose biosynthesis. For instance, clearance CBIs have been used to garner guilt by association logic for co-clearance of CESA and CESA-interacting proteins such as GFP:KOR1 (KORRIGAN1, Robert et al., 2005) and GFP:CSI1 (CELLULOSE SYNTHASE INTERACTING1, Bringmann et al., 2012). Although this alone fails to prove association, it adds to the usefulness of CESA clearance compounds outside of studying CESA behavior.

## STOPPING OF CESA PLASMA MEMBRANE MOBILITY

The second CESA response phenotype is increased accumulation and cessation of CSC movement in the plasma membrane (Herth, 1987; DeBolt et al., 2007b). Currently this group consists of one compound, DCB (2,6-dichlorobenzonitrile; **Figure 1** – Group 2). DCB, another synthetic herbicide marketed since the 1960s, is second only to isoxaben as an experimental probe (Sabba and Vaughn, 1999).

2,6-Dichlorobenzonitrile exhibits a broad range of activity on species with terminal complexes, regardless if it is in lower species with a linear-complex or the rosette form found in higher plants (Mizuta and Brown, 1992; Orologas et al., 2005; DeBolt et al., 2007b). This suggests that DCB targets cellulose synthesis in a range of organisms, however, in species with linear-terminal complex such as the red alga *Erythrocladia subintegra*, treatment resulted in disappearance from the plasma membrane (Orologas et al., 2005). An early clue toward the molecular function of DCB was discovered when an DCB analog was found to bind a small protein of 12 or 18 kDa from suspension-cultured tomato cell extracts or cotton fiber extracts, respectively (Delmer et al., 1987). The amount of bound protein seemed to increase significantly at the onset of secondary cell wall synthesis in cotton fibers. Recently, the same DCB analog target using a biochemical approach was identified in hybrid aspen (*Populus tremula* × *tremuloides*) and found to be MAP20 (Rajangam et al., 2008). Microtubule-associated proteins (MAPs) have been shown to bind to microtubules (MTs) and play a role in the synthesis of the secondary cell walls in *Arabidopsis*, as the FRAGILE FIBER1 (FRA1) and FRA2 kinesin-like proteins influence cellulose microfibril patterning in the inner wall of interfascicular fibers (Zhong et al., 2002; Burk et al., 2007). *In lieu* of this data, Wightman et al. (2009) used the confocal technique FLIP (fluorescence loss in photobleaching) to observe that DCB treatment also slowed CSC tagged YFP:AtCESA7 needed for secondary wall deposition. This could indicate that MAPs are necessary for primary and secondary cell wall development.

## MODIFYING CESA TRAJECTORY

The third disruption mechanism of the CSC is co-disturbance of both CESA and cortical MT. The molecular rail hypothesis (Giddings and Staehelin, 1988), suggests that MTs act as a guidance mechanism for the CSC. Using dual labeled CESA and MT reporter lines this can be visualized in real time showing that coincident MT and CESA arrays are often perpendicular to the axis of elongation during expansion (Paredez et al., 2006). Interestingly, when MTs are pharmacologically depolymerized via the drug oryzalin, YFP-CESA6 plasma membrane trajectory (organization of direction) but not velocity was altered (Paredez et al., 2006; DeBolt et al., 2007a). The velocity or positional change over time suggests that the CSC is moving the plasma membrane while making cellulose (Paredez et al., 2006). Interpretation of this evidence implies that the force of glucan chain polymerization is responsible for CSC movement in the plasma membrane rather than MTs or MT motor proteins.

Within this group of compounds that we clustered based on modifying CESA trajectory, some do not cause depolymerization of MTs. These compounds were identified in forward chemical genetic screens for compounds affecting cell wall synthesis and morphology (DeBolt et al., 2007a; Yoneda et al., 2007). The first of two compounds is a coumarin derivative, named morlin (7-ethoxy-4-methyl chromen-2-one; **Figure 1** – Group 3). Analysis using live-cell imaging of fluorescently labeled MAP4 (microtubule-associated protein-4) revealed that morlin caused a defect in cytoskeleton organization that actually hyper-bundled the MTs. The CESA arrays were also disorganized compared to control cells, but instead of clearing CESA from the plasma membrane, morlin treated cells displayed reduced CESA velocity that was independent of MTs. Likewise, in a similar screen looking for a swollen cell phenotype in tobacco BY-2 cells, cobtorin (4-[(2-chlorophenyl)-methoxy]-1-ntirobenzene) (**Figure 1** – Group 3) was identified as a potent compound that distorts the behavior of both CESA and MT (Yoneda et al., 2007, 2010), not dissimilar to that of morlin. It was further discovered that pectin methylation mutants could decrease the effectiveness of cobtorin. Further elucidation of the feedback between CSCs and MTs in multiple cell types and growth phases will provide important data for pinpointing the mechanisms of cell shape acquisition and it is evident that small molecule inhibitors will be valuable tools in this endeavor.

## CHEMICAL GENETICS: RESISTANCE OR HYPERSENSITIVITY LOCI

As additional chemical screens are completed and new compounds are identified that target the cell wall, it is imperative that they be followed up with forward resistant or hypersensitive screenings for detection of new molecular players in cell wall biosynthesis. An example of a resistant screen was recently performed for the quinoline derivative, quinoxyphen. The resistant locus for this drug was determined through a map-based approach in *Arabidopsis* to *CESA1* (Harris et al., 2012). The quinoxyphen-resistant mutant also shows a growth phenotype only slightly reduced to that of wild-type, thus representing a viable, non-conditional mutation in CESA1. This screen followed the logic generated in the screen for isoxaben-resistant (*ixr*) mutants (Heim et al., 1989). Here, the loci conferring resistance to isoxaben were mapped to *cesa3*^*ixr1*^ and *cesa6*^*ixr2*^ (Scheible et al., 2001; Desprez et al., 2002). The mutations conferring resistance to isoxaben and quinoxyphen are not found near the putative active site for CESA1, CESA3, or CESA6. Rather, the resistance conferring mutations are located in the C-terminal TMD of these gene products. The TMD region mutations individually caused a reduction in the degree of crystallinity created by the inter and intra chain hydrogen bonding between glucan chains comprising cellulose in the mutant plants (Harris et al., 2012). In turn, this resulted in greater conversion of the cellulose within the biomass to fermentable sugars. This information may prove to be a significant finding for the lignin-cellulosic biofuel field. Further studies are needed to determine the usefulness of such mutations under field situations and to determine the biochemical rationale for such mutations.

While no resistant mutant has been identified for AE F150944 or CGA 325’615, a forward genetics resistance screen to thaxtomin A in *Arabidopsis* identified the gene *TXR1* (*THAXTOMIN RESISTANCE-1*) that encodes a novel small protein most likely involved in the regulation of a transport mechanism and thus may provide resistance by reducing plant uptake of thaxtomin A (Scheible et al., 2003). Specifically, N- and C-terminal GFP fusions to TXR1 were localized in the cytoplasm of tobacco leaf protoplasts, suggesting that the protein acts as a cytosolic regulator of a membrane protein rather than being a permanent component of a transporter complex. The focus of future studies will be to determine whether the GFP fusions correctly reflect the localization of TXR1 and with which proteins TXR1 interacts (Scheible et al., 2003). The identification of mutants of this nature are good examples of how resistance to a small molecule is not always target-site based and may occur by preventing the drug from reaching the site of action via metabolism, reduced uptake, or altered translocation. In the future, if forward resistance screens are successful toward AE F150944 or CGA 325’615, it will be interesting to learn whether the resistance loci map to CESA or to new molecular players in cellulose biosynthesis.

An example of an opposite screen, hypersensitivity, was performed using an EMS-mutagenized *Arabidopsis* population to the compound flupoxam (1-[4-chloro-3-[(2,2,3,3,3-pentafluoro-propoxy)methyl]phenyl]-5-phenyl-1H-1,2,4-triazole-3-carboxamide) (Austin et al., 2011). Flupoxam is a characterized CBI as has not been examined using live-cell imaging (Hoffman and Vaughn, 1996). Two mutants were identified through the use of next-generation-mapping technology as *flupoxam hypersensitive 1* and *2* (*fph1*, *fph2*). The loci were identified as *ECTOPIC ROOT HAIR3* (*ERH3*) for the *fph1* locus and *OLIGOSACCHARIDE TRANSMEMBRANE TRANSPORTER *(*OST3/OST6*) for the *fph2* locus. Neither *ERH3/FPH1* nor *OST3/OST6/FPH2* encoded known cell wall biosynthetic enzymes and consequently this screen identified potential regulators of cell wall composition (Austin et al., 2011).

Resistant- or hypersensitive-mutants to the compounds that perturbed the parallel alignment of pre-existing cortical MTs and nascent cellulose microfibrils have not been decoded for morlin however, success has been made with cobtorin. The target proteins are likely to have an important role in the relationship between MTs and microfibrils. Yoneda et al. (2007) employed the *Arabidopsis* FOX hunting library, an activation tagging technology that makes use of full-length cDNAs that create gain-of-function mutants. From approximately 13,000 FOX lines, three cobtorin-resistant lines were identified and mapped to a lectin family protein, a pectin methylesterase (AtPME1) and a putative polygalacturonase (Yoneda et al., 2010). This study goes on to show some important features of pectin in relation to the formation and orientation of cellulose microfibrils, which depends on the methylation ratio of pectin and its distribution (Yoneda et al., 2010), which has recently been experimentally explored by ^13^C solid-state magic-angle-spinning NMR (Dick-Perez et al., 2011).

As described, identification of drug targets linked to novel mechanisms of action can delineate information that is difficult to obtain via classical reverse genetics and are powerful tools in elucidating the dynamics of plant cell walls. It is fully expected that additional inhibitory mechanisms exist and academia and industry are keenly waiting for them to be identified. We apologize to the authors of other papers that have provided significant information to this field, as it was not possible to discuss the entire range of chemical agents and experimental results.

## Conflict of Interest Statement

The authors declare that the research was conducted in the absence of any commercial or financial relationships that could be construed as a potential conflict of interest.

## Abbreviations

CBI: cellulose biosynthesis inhibitor
CESA: cellulose synthase A
CSC: cellulose synthase complex
DCB: 2,6-dichlorobenzonitrile
GFP: green fluorescent protein
MAP: microtubule-associated protein
MT: microtubule
TMDs: transmembrane domains
YFP: yellow fluorescent protein.
